# Comparison of two different types of heat and moisture exchangers in ventilated patients

**DOI:** 10.4103/0974-2700.55327

**Published:** 2009

**Authors:** Syed Moied Ahmed, Jyotsna Mahajan, Abu Nadeem

**Affiliations:** Department of Anaesthesiology and Critical Care, JN Medical College, Aligarh Muslim University, Aligarh, India

**Keywords:** Bacterial filters, heat and moisture exchangers, ventilator-associated pneumonia

## Abstract

**Study Objectives:**

To compare the efficacy of two different types of Heat and Moisture Exchangers (HME filters) in reducing transmission of infection from the patient to ventilator and vice versa and also its cost effectiveness.

**Design:**

Randomized, controlled, double blind, prospective study.

**Patients and Methods:**

60 patients admitted to the ICU from May 1, 2007 to July 31, 2007 of either sex, age ranging between 20 and 60 years, requiring mechanical ventilation were screened for the study. Following intubation of the patients, the HME device was attached to the breathing circuit randomly by the chit-in-a box method. The patients were divided into two groups according to the HME filters attached.

**Results:**

Both the groups were comparable with respect to age and sex ratio. In Type A HME filters, 80% showed growth on the patient end within 24 h and in 27% filters, culture was positive both on the patient and the machine ends. The organisms detected were *Staphylococcus aureus, Escherichia coli* and *Pseudomonas aeruginosa* and co-related with the endotracheal aspirate culture. After 48 h, 87% filters developed organisms on the patient end, whereas 64% filters were culture positive both on the patient and the machine end. In Type B HME filters, 70% showed growth on patient's end after 24 h. Organisms detected were *S. aureus, E. coli, P. aeruginosa* and Acinetobacter. Thirty percent of filters were culture negative on both the patient and machine ends. No growth was found on the machine end in any of the filters after 24 h. After 48 h, 73% of the filters had microbial growth on the patient end, whereas only 3% filters had growth (*S. aureus*) on the machine end only. Seven percent had growth on both the patient as well as the machine ends. The microorganisms detected on the HME filters co-related with the endotracheal aspirate cultures.

**Conclusion:**

HME filter Type B (study group) was significantly better in reducing contamination of ventilator from the patient as compared to Type A (control group), which was routinely used in our ICU. Type B filter was found to be effective for at least 48 h. This study can also be applied to patients coming to emergency department (ED) and requiring emergency surgery and postoperative ventilation; and trauma patients like flail chest, head injury etc. requiring ventilatory support to prevent them from acquiring ventilator-associated pneumonia (VAP).

## INTRODUCTION

Nosocomial pneumonia (NP) or hospital-acquired pneumonia is defined as pneumonia occurring more than 48 h after hospital admission and excluding any infection that is incubating at the time of hospital admission. Incidence of NP in general ICU patients varies between 8 and 20%. This risk is about 10 folds higher in ICU patients treated with endotracheal intubation and mechanical ventilation as compared to patients with no respiratory therapy devices.

Nosocomial pneumonia is associated with a high mortality rate of around 30%.[1]

Patients with ventilator-associated pneumonia (VAP) have a longer duration of mechanical ventilation, ICU stay and hospital stay. It is also associated with increased hospital charges.[2]

Incidence of VAP increases by 1 to 3% per day of intubation, and it increases hospital stay by 4-9 days and contributes to 15% of hospital deaths.

To add to this problem, successful treatment of patients with NP is still a challenging task for intensivists. No concensus has still been reached regarding issues like optimal antimicrobial therapy or duration of treatment. Controversy still exists regarding preference for monotherapy or combination therapy and also use of endotracheal or aerosolized antibiotics as either sole or adjunctive therapy.

In the face of such a scenario, prevention of VAP becomes a dire need for the success of emergency and intensive medicine in order to decrease morbidity and mortality, duration of hospital stay and health care costs. This is especially important in patients who already have an anticipated prolonged ICU stay, for example polytrauma patients with head injury and major chest and abdominal injuries, requiring ventilatory support. Any superadded iatrogenic infections in such patients, worsens their prognosis along with increasing hospital stay and economic burden.

There are several recommendations for prevention of infections. Changing of tubing regularly and sterilization of the tubing may be expensive, time consuming and environmentally unfriendly. Further, some studies showed that these methods are not always effective. In recent years, use of filters has become very popular in intensive care since this is user friendly, effective and economical.

As the patient is the most likely source of initial contamination of a circuit, probably the most logical place to position the filter is as near to the patient as possible. The ‘ideal patient end’ filter should totally retain contaminated liquids, have high airborne microbial removal efficiency, a low resistance during clinical use and be able to be left in place during nebulization. In addition, the device should also act as a heat and moisture exchanger (HME). Currently, there are a whole range of different devices that have been proposed for use at the ‘patient end’ of a breathing circuit and it is extremely difficult to know whether or not a device possesses these attributes.

The aim of this study was to compare the efficacy of two different types of HME filters in reducing transmission of infection from the patient to ventilator and vice versa and its cost effectiveness.

## PATIENTS AND METHODS

A randomized, controlled, double blind, prospective study was conducted in the Intensive Care Unit of a tertiary care hospital on medico-surgical patients (including trauma cases). Sixty consecutively admitted patients of either sex and age ranging between 20 and 60 years who required mechanical ventilation for an anticipated period of more than 48 h were selected for the study. The study included patients who had to be urgently intubated in the emergency room, cardiac unit or high dependency unit of the hospital or who could not be extubated after an emergency surgery and were then shifted to the ICU for further management. The study was approved by the departmental board of studies and written consent was obtained from the patient's relatives.

Following intubation of the patients, the HME device was attached to the breathing circuit randomly. The HME device routinely used in our center (Hygrobac ‘S’ type) was taken as the control and compared with a newer variety (Intersurgical type). The randomization was done by the chit-in-a box method. The patients were divided into two groups according to the HME filters attached.

**Figure 1:**
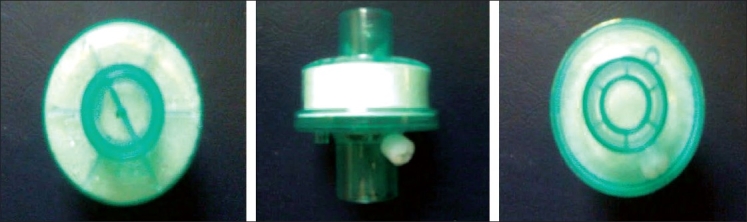
The pictures show the “Hygrobac S HME Filter” used for the control group

Control group (*n* = 30) – HME filter (Type A, Hygrobac ‘S’, Figure 1) attached to the breathing circuit of the ventilator

**Figure 2:**
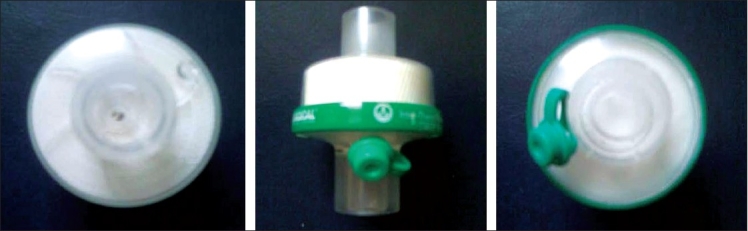
The pictures shows the intersurgical HME filters used for the study group

Study group (*n* = 30) – HME filter (Type B, Intersurgical, Figure 2) attached to the breathing circuit of the ventilator

Patients who were newly admitted in the ICU and not on any antibiotics were administered empirical antibiotic therapy as per the ICU protocol, which included broad spectrum antibiotics and any specific antibiotic pertaining to the site of infection, e.g. for pelvic abscess, meningitis etc. Admitted patients in the ICU who were already on antibiotics, and required intubation later in the course of their illness were not included in the study.

Those patients who were extubated within 48 h of intubation and those in whom the HME filters were soiled with any visible secretions (blood, sputum), were excluded from the study and a new patient was selected in his/her place in both the conditions.

The following parameters were recorded:

- Using aseptic precautions, specimens were taken for culture from the following sites:
  1. Endotracheal aspirate immediately after intubation
  2. Patient end and machine end of the HME filter after 24 h
  3. Patient end and machine end of the HME filter after 48 h
    The specimens were sent to the Microbiology Department of the hospital for culture and the antibiogram was obtained after 72 h of incubation.
- The changes if any in the Peak Inflation Pressure (PIP) were recorded from the ventilator monitor readings with and without the filters attached to the circuit:
  1. Immediately after intubation
  2. After 24 h of intubation
  3. After 48 h of intubation

The change in the PIP following attachment of the HME filters to the circuit at 24 and 48 h would indirectly indicate obstruction or blockage of the filter due to the soiling with secretions, which otherwise was not visible.

All the HME filter devices were routinely changed after 48 h of ventilation as per the ICU protocol. Other measures for reducing incidence of VAP, such as use of semi-recumbent positioning for hemodynamically stable patients, regular aspiration of subglottic secretions and stress ulcer prophylaxis were followed for all patients.

### Statistical analysis

Fischer's test was applied to analyse the difference between study and control groups for growths on patient end only and growths on patient and machine ends. *P* value less than 0.05 was considered statistically significant.

Demographic data and type of organisms found on the HMEs were analysed using paired *t* test. Again *P* value less than 0.05 was considered significant.

HME devices discarded in both the groups were evaluated with Fischer's test.

## RESULTS

Both the groups were comparable with respect to age and sex ratio [Table 1].

**Table 1:** Demographic characteristics of subjects

	Control group	Study group
Number	30	30
Age (mean); years	36	37.5
Male / Female	19 / 11	18 / 12
Medical patients	20	21
Stroke	3	2
CHF	4	5
GB syndrome	1	0
COPD	5	7
Snake/insect bite	2	3
Hepatic encephalopathy	1	0
Tubercular pleural effusion	3	1
Organophosphorus poisoning	1	3
Surgical patients	10	9
Gunshot/stab wound abdomen	0	1
Blunt trauma chest with flail chest	1	1
Blunt trauma abdomen	2	0
Head injury	2	3
Intestinal obstruction	3	2
Perforation peritonitis	2	2

After 24 h, 53.3% (16) filters of the Control group (Type A) showed growth only on the patient end and 26.7% (8) filters showed growth on both the patient as well as the machine end. This implies that a total of 24 (80%) filters were contaminated on the patient end out of which 16 (53%) were unable to stop contamination of the other end of the circuit. In the Study group (Type B), 70% (21) of the filters showed positive cultures at the patient end only and none of the filters had growth on both the ends, meaning that all of these filters were able to prevent contamination of the other end of the filter and protect the circuit [Table 2].

**Table 2:** Comparison of microbial growth in subjects

Growth on	Control group		Study group		
			
	24 h	48 h	24 h	48 h	
Patient end only	16	7	21	20	*P* > 0.05
Machine end only	0	0	0	1	
Both patient and machine ends	8	19	0	2	*P*< 0.05
Sterile on both ends	6	4	9	7	
n	30	30	30	30	
Total positive cultures on patient end	24 (16 + s8)	26 (7 + 19)	21 (21 + 0)	22 (20 + 2)	*P* > 0.05

After 48 h, 23.3% (7) of the filters were culture positive only at the patient end in the Control group (Type A), whereas 63.3% (19) filters were culture positive both on the patient and the machine end. That is about 87% (26) filters got the contamination at the patient end and only 7 filters were able to protect the circuit from getting contaminated. In comparison, 66.7% (20) filters of the Study group (Type B) had microbial growth on the patient end only, whereas a mere 6.6% (2) cases had growth on both the patient as well as the machine ends. Another 3.3% (1) filters had growth (*S. aureus*) on the machine end only and they had sterile endotracheal aspirates. In all other cases, the micro-organisms detected on the HME filters co-related with the endotracheal aspirate cultures. The organisms found were *S. aureus, Streptococcus pneumoniae, E. coli, Klebsiella* and *P. aeruginosa* in both the groups. In addition, the study group (Type A) had one case infected by Acinetobacter species as well [Table 3].

**Table 3:** Types of microorganisms found in culture

Microorganism	Control group			Study group		
		
	0 h (at intubation)	24 h	48 h	0 h (at intubation)	24 h	48 h
*Staphylococcus aureus*	8	12	13	8	13	14
*Escherichia coli*	5	9	10	4	6	6
*Pseudomonas aeruginosa*	0	1	3	0	2	2
*Acinetobacter* species	0	0	0	0	0	1
Total number of positive cultures *	13 (10 Med + 3 Surg)	22 (16 + 8)	26 (7 + 19)	12 (9 Med + 3 Surg)	21	23 (20 + 1 + 2)

* TOTAL NUMBER OF POSITIVE CULTURES = NUMBER OF POSITIVE CULTURES AT PATIENT END ONLY + NUMBER OF POSITIVE CULTURES AT MACHINE ENDS + NUMBER OF POSITIVE CULTURES AT BOTH PATIENT AND MACHINE ENDS.

In the Control group (Type A) 13.3% (4) filters did not show any organism on either the patient or the machine end at the end of 48 h. Similarly, 23.3% (7) filters in the Study group (Type B) had sterile cultures. The endotracheal aspirates of all these patients were also culture negative.

After 24 h the number of filters showing microbial growth on the patient end was comparable in both the groups [80% (24 filters) in Control group versus 70% (21 filters) in Study group]. However, filters having growth on both the ends (patient and machine) were significantly less in the Study group [26.7% (8 filters) in Control group versus 0% (none) in Study group; *P* < 0.05].

Further, after 48 h the number of filters showing microbial growth on both the patient and machine end was significantly more in the Control group than in the Study group (63.3% in control group vs 6.6% in study group; *P* < 0.05) though the microbial infection in the patient end was similar in both the groups [86.7% (26 filters) in Control group vs 73.3% (22 filters) in study group] [Table 2].

The rise in PIP was lesser in the Study group as compared to the Control. As a result the number of devices discarded due to raised PIP was lesser in the Study group [Table 4].

**Table 4:** Comparison of HME devices discarded between groups

HMEs discarded due to	Control group		Study group	
		
	0-24 h	24-48 h	0-24 h	24-48 h
Visible soiling	3	2	4	2
Raised PIP	2	3	0	1
Total	5	5	4	3

Fischer's test was applied to analyse the observations and a statistically significant difference was found between both the groups in having microbial growth on both the patient and machine ends of HME devices after 24 and 48 h. This implies that the Study group (Type B HME devices) was better in preventing the transmission of infection from the patient to the ventilator and was effective for at least 48 h.

The number of HME devices discarded due to rise in PIP was also lesser in the Study group, meaning that Type B filters were also better in humidification of inhaled gases.

## DISCUSSION

There is now a wealth of clinical papers demonstrating that breathing circuits used in ICU can become contaminated during use. Contamination of breathing systems, which can arise either via medical gases or through the patient, is a major source of ventilator acquired pneumonia (VAP).

Ventilator acquired pneumonia (VAP) is an expensive, time consuming and potentially fatal complication of respiratory therapy. Various prophylactic measures have been suggested for prevention of VAP, e.g., proper patient positioning, use of oscillating beds, aspiration of subglottic secretions, ventilator circuit management, stress ulcer prophylaxis etc.[3,4]

Recently, use of bacterial and viral filters in ventilator circuits has become a very popular and cost-effective means of reducing infection secondary to mechanical ventilation. It is now well known that the use of humidified gases in ventilated patients is associated with better preservation of ciliary motion of the respiratory tract and lesser chances of infection. Humidification of gases can be done by either active (Heat Humidifiers) or passive (HME devices) methods. Heat Humidifiers use an external humidification source for providing moist gases to patients whereas HMEs use patient's own heat and moisture for the same. It has been found in various studies that the technique of humidification offers no benefit in mortality rates or rate of development of VAP.[5,6,7] Use of HME devices considerably reduces nurses' workload and financial demands and is thus more user friendly than heat humidifiers.

In this study, cultures were taken from three sites. Endotracheal aspirate soon after intubation on culture indicated the prior presence of any respiratory tract infection. Taken from the patient end of the device after 24 h, it indicated the spread of infection from the patient towards the ventilator. Cultures taken from the machine end of the device determined the efficacy of the filter in preventing the transmission of microorganisms from the patient to the ventilator circuit. They were taken serially after 24 and 48 h to determine the duration for which the device could be used.

Correlation between the microorganisms detected in the endotracheal aspirates and those found on the HME device indicate that the contamination was obtained from the patient and spread towards the ventilator. The only case which had a positive culture on the machine end with a negative culture on the patient end probably got the contamination from the ventilator rather than the patient, since the endotracheal aspirate of this patient was also sterile.

We found that many of our patients had chest infection at the time of intubation, more so in medical rather than surgical patients. This is possible as most of these patients were already hospitalized and were admitted to the ICU from the wards. Therefore, it is quite likely that they already had acquired nosocomial infections, especially if they had also been immunocompromized due to their prolonged illness. This fact is also supported by the observation that the endotracheal aspirates of majority of patients yielded *S. aureus* and *E. coli*, which are mainly responsible for hospital acquired infections. The surgical patients who were brought to ICU from inadequate recovery from anesthesia could also have acquired such infections because of severe illness especially in cases of intestinal obstruction and peritonitis, which lead to severe derangements in body's homeostatic mechanisms.

It is very difficult to remove biasness in any study. However, we tried to make our study bias-free by making the observer blind about the collected samples. The samples were collected by the ICU doctors and were coded and sent to the Microbiology Department of the Hospital, where cultures were performed. The personnel involved in identifying the growth were not aware of the site from where the specimen was taken (endotracheal, patient end or machine end) and the time when the specimen was taken (just after intubation, after 24 h or 48 h).

HME devices studied earlier have claimed to be of equal efficacy, but our results differ as there was a significant difference between the efficacy of the two types of devices studied. The Intersurgical HME devices proved to be far more superior compared to Hygrobac S devices as infection rate at both ends was only 6.6% after 48 h compared to 26.6% in the latter after only 24 hours.

The limitation of our study was that the colony count (quantitative analysis) of the microbial growth obtained on the HME devices was not done. Therefore the significance of the presence of growth could not be evaluated. Another limitation was that since the culture reports were obtained after 72 h of incubation, it could not be estimated during the study how long the devices would be effective and necessitate change of the device. We changed the devices after 48 h as per the recommendation of the previous studies. In the Control group more than 60% of the filters were infected on both the sides. This endangered our circuits. However, the circuits used with Study group filters were privileged. Hence, further studies would be required to assess the actual duration for which this device could be used and also its cost effectiveness. We presume that the Intersurgical HMEs are more cost-effective than Hygrobac S HMEs as there is lesser contamination of the ventilators with the use of the former till 48 h and lesser number of them were discarded due to visible soiling and rise in PIP. Also the market price of both the filters is nearly the same, with Intersurgical HMEs costing only about Rs. 10 more to the patient than Hygrobac S HMEs. But since we do not know the exact duration for which they can be used effectively, any quantitative assessment of cost-effectiveness cannot be made.

Rise in PIP was taken as a measure of humidifying ability of the device. A measure of the difference of temperature at both ends of the device along with a PIP rise could have served as a better guide of the same.

Another deficiency was that we studied only the transmission of contamination from the patient towards the ventilator but not any increase in the incidence of VAP. Therefore, further scope remains to study the effect of this microbial transmission on the change in incidence of VAP, if any using more clinical and microbiological parameters.

## CONCLUSION

HME filter Type B (Study group, Intersurgical) was significantly better in reducing contamination of ventilators from the patient as compared to Type A (Control group, Hygrobac S), which was routinely used in our ICU.

Further, it was also found to be effective for at least 48 h, for both medical and surgical patients, including polytrauma cases.
